# Dental Pain in Care Homes: Is It a Phenomenon? A Systematic Review of the Literature

**DOI:** 10.3390/geriatrics7050103

**Published:** 2022-09-26

**Authors:** Pat Schofield, Nicole Thomas, Ewen McColl, Robert Witton

**Affiliations:** 1 Clinical Nursing, School of Nursing & Midwifery, University of Plymouth, Plymouth PL4 8AA, UK; 2 e-Health, School of Health Professions, Plymouth PL4 8AA, UK; 3 Clinical Dentistry, Peninsula Dental School, University of Plymouth, Plymouth PL4 8AA, UK; 4 Community Dentistry, University of Plymouth, Plymouth PL4 8AA, UK

**Keywords:** dental pain, dementia, care homes

## Abstract

Background: Evidence suggests that 80% of residents living in nursing homes have moderate to severe pain, could dental causes be an under reported contributory factor. The evidence suggests that this is an under-researched area. Our project aims were to explore and consolidate the current literature and conduct some stakeholder groups with care home managers and dentists. Our stakeholder group will be reported elsewhere. Methods: We used the SPIDER framework to set out key search terms. Which included “dementia” OR “cognitively-impaired” OR “carehome residents” AND “dental pain” OR “oralfacial pain” OR “mouth pain” AND “pain assessment” OR “pain identification”. A literature search was carried out on 8 and 9 March 2022 in the electronic databases: Cochrane, PubMed, Medline, Dental & Oral Sciences Source, CINAHL, Global Health, SocINDEX, Ovid (Medline) and Scopus. Restrictions were placed on dates and language (2012–2022 and English only). Results: The search yielded 775 papers up to the year 2020. After screening and exclusion, we were left with five papers: four quantitative and one qualitative. Conclusions: This review demonstrates that there has been very little research into oral health and/or dental pain in adults with dementia. Furthermore, the recommendations have yet to be taken forward. Identifying pain in older adults with dementia remains challenging. There is a need to develop an algorithm in conjunction with care home staff and dental practitioners in order to identify and address the pain associated with dental disease in adults with dementia.

## 1. Background

The Western world is facing huge challenges in coming years. The World Health Organization has identified the ageing global population as an important medical and social problem [1]. People are living longer, and the population is ageing fast. In 2015, the UK population over the age of sixty was 9 million, and by 2050 it is anticipated that this population globally will exceed 2 billion [2]. Globally, there are currently 145 million people aged over 80 years and by 2050 there will be that many in China alone [3]. By 2050 we will see 80% of the over-80 population living in low to middle income countries [2,4].

In the UK and Europe, we know that better health systems have improved life expectancy but issues related to housing, geography and social deprivation continue to impact upon health and well-being, as we are seeing amongst the post-war baby boomers reaching their sixties [5,6]. However, whereas people are living longer, this does not necessarily equate to better health. Modern living has seen an increase in smoking, alcohol use and inactivity, which all influence comorbidities [7]. A number of comorbidities are seen in the older population including: frailty, falls and cognitive decline. Although the World Health Organization does not highlight chronic pain amongst these syndromes, our recent systematic review identified three specific pain syndromes in this population (Back (16 studies), leg, knee or hip (16 studies, other joints 5 studies) [8] and the updated version confirms these pain syndromes [9].

The International Association for the Study of Pain [10] defines chronic pain as that which persists beyond the expected healing time and suggests that it often has no identifiable cause and it is often incurable [10,11]. The expectation being that the individual will have to learn to live with ongoing pain and this has resulted in the introduction of cognitive behavioural methods to support the self-management of pain. Living with chronic pain is challenging. We know from our recent work that 40% of the older population living in the community have poorly controlled chronic pain. This figure is purported to increase to 80% amongst those living in care homes [12]. As discussed previously, common chronic pain syndromes are well documented within the care home setting, but what about dental pain? Our research group asked the question as to whether dental pain could be one of the unidentified pain syndromes that could explain such a high incidence of poorly controlled pain identified within the literature.

A recent systematic review of behavioural interventions targeting pain in people living with dementia found that both pain and challenging behaviour are highly prevalent in this group [13]. The review cited multiple studies showing pain and challenging behaviour were correlated. The paper concludes that pain, especially in non-communicative dementia patients, can cause challenging behaviour. Equally, treatment of pain can improve any challenging behaviour that people living with dementia may present, as well as improve their quality of life [13]. A recent study by Conway [14], demonstrated poor quality or no pain assessment practice in care homes in Kent, and equally identified a high number of assaults upon staff associated with working in environments where there are high numbers of dementia patients. Less research is available on the prevalence and management of pain in ‘community-dwelling’ older adults with dementia [15].

The Adult Dental Health Survey (ADHS) revealed that over half of older people (aged over 60) are maintaining some, if not all, of their natural dentition, i.e., teeth [16]. Furthermore, we are increasingly likely to see older adults and those with dementia having much more complex dental health needs, along with digital dentistry and other more technical requirements such as crowns and implants, including rehabilitation with tilted or axial implants [17,18,19], However, although people are ‘keeping their teeth’ to old age, there is an increased prevalence of tooth decay and dental pain in the older population—with poor dentition increasing more rapidly in those aged 80 and over [16]. One local Joint Strategic Needs Assessment in the UK [20] estimated that 20% of older adults in the South East and Costal Region have active tooth decay and 25% of older adults suffer from severe gum disease. Equally, 7% of older adults reported they suffered from dental pain at the time of the survey. For patients living with dementia there is an increased risk of deteriorating oral health—and thus the potential to experience dental pain—brought about by factors such as poor monitoring of oral hygiene and the effects of polypharmacy [21]. It has been found that dental pain is frequently undetected in people living with dementia, for example, in a US study, Cohen-Mansfield and Lipton [22] found that up to 60% of their sample of people living with dementia had a dental condition that was likely to cause pain.

Very little data exists on the prevalence of dental pain in people living with dementia in the UK, but there is a growing guidance on maintaining oral health for older people living with dementia in residential settings (e.g., Pearson and Chalmers, 2004 [21]. One key recommendation is that training of nursing staff, allied health workers as well as carers (both formal and informal) in oral hygiene monitoring and assessment strategies (including the use of oral health screening tools) can improve access to dental treatment for, and the management of oral diseases and conditions in, adults living with dementia in care settings [21]. Also, effective pain assessment tools for identifying pain in older people living with dementia, which can be administered by healthcare professionals (e.g., see Herr et al., 2006 [23]) and informal carers/family (e.g., see Buffum et al., 2007 [24]), do exist.

Thus far, we can see that when people living with dementia—whether in the community or in care environments—frequently experience unmanaged pain. The poor oral health status and dentition associated with old age (alongside other factors specific to people living with dementia such a polypharmacy) may also increase the likelihood of people living with dementia experiencing dental pain. Studies have shown that it is possible to identify and manage pain in people living with dementia, as do oral health screening tools for people living with dementia, yet dental pain is frequently undetected.

## 2. Evidence Synthesis

The methodological quality of the included systematic review will be assessed with the Preferred Reporting Items for Systematic reviews and Meta-Analyses (PRISMA) State-ment (Supplementary Materials S1). Due to the dearth of literature identifying the prevalence of dental pain in people living with dementia in the UK, we used a systematic review approach to look at the dental pain assessment tools used for this population. In particular, we wanted to include any innovations that may have been developed to assess dental pain. Additionally, it is important for decision makers to understand the possible barriers associated with using such tools and qualitative evidence can provide that understanding [25]. Therefore, we did not exclude any type of evidence on the grounds of its methodology and included qualitative studies to explore the experiences of those (informal and formal carers) assessing dental pain in this population.

In order to capture both quantitative and qualitative studies, we used the SPIDER framework to set out key search terms. Search terms included “dementia” OR “cognitively-impaired” OR “carehome residents” AND “dental pain” OR “oralfacial pain” OR “mouth pain” AND “pain assessment” OR “pain identification”. A literature search was carried out on 8th and 9th March 2022 in the following electronic databases: Cochrane, PubMed, Medline, Dental & Oral Sciences Source, CINAHL, Global Health, SocINDEX, Ovid (Medline) and Scopus. Restrictions were placed on dates and language (2012–2022 and English only). No restrictions were placed on the design or methodology to maximise the inclusion of appropriate articles.

Articles were uploaded to Rayyan [26], duplicates were removed and the titles and abstracts screened independently by two researchers (NT, PS). Using the decision tagging function of Rayyan, ‘yes’, ‘no’ and ‘maybe’ were tagged to each paper. Disagreements were reviewed and non-relevant articles were removed before screening full texts. If the paper did not relate specifically to a dental pain assessment tool or to people living with dementia or cognitive impairment, the article was excluded.

The Critical Appraisal Skills Programme tool [27] was used to assess the quality of the studies, dependant on their design (cohort, case–control or qualitative).

## 3. Results

### 3.1. Study Selection

**Figure 1:**
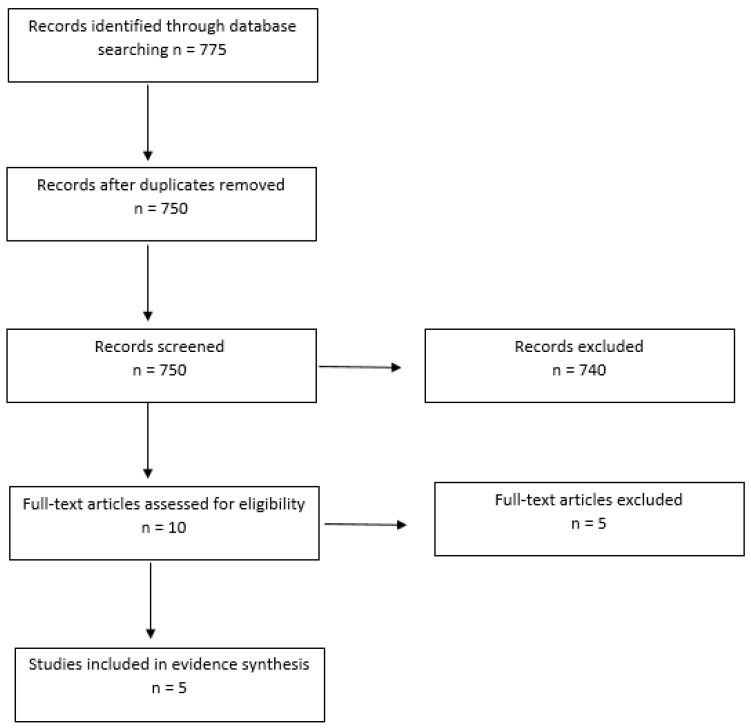
Flow chart of the literature selection for evidence synthesis.

The search yielded 775 papers up to the year 2020. After duplicates had been removed, 750 papers remained. The titles and abstracts of the remaining papers were screened, leading to a further 740 papers being excluded due to not meeting the criteria. The remaining 10 full-text articles were screened to examine eligibility for inclusion and a further five papers were removed, with five being included for the evidence synthesis: four quantitative papers (correlational and or systematic reviews) and one qualitative paper (cross-sectional exploratory study). Figure 1 provides a flow chart of the literature selection.

### 3.2. Dental Pain Assessment Tool Studies

The quantitative papers included the evaluation of three adapted pain assessment tools for oral-facial pain assessment in people with dementia.

A study by Toxopeus et al. [28] used the Mobilization–Observation–Behaviour–Intensity–Dementia (MOBID) Pain Scale and adapted it to an orofacial MOBID scale [28]. The aim of the study was to determine the reliability of using this tool to identify dental pain separate from dementia-associated behaviour. The study had a sample of 11 dementia residents of a nursing home. The study used 12 elderly-care dentists to observe 11 video fragments of carers carrying out oral hygiene for the 11 dementia residents (10 female, 1 male). The observer scores were based on at least one box ticked, per fragment, per session, for each category (orofacial pain/discomfort-related behaviour, dementia-related behaviour or both) that was identified as Choice I, II or II. Choice I was if an observer ticked at least one box for orofacial pain/discomfort-related behaviour. Choice II was dementia-related behaviour and Choice III was both categories. An arbitrary agreement threshold at ≥66.6 for substantial agreement on dementia behaviour, dental-pain behaviour or both was used.

Two studies evaluating the Orofacial Pain Scale for Non-Verbal Individuals (OPS-NVI) had a total sample of 432 of dementia sufferers located in the UK and the Netherlands [29,30]. Another study evaluated the “chewing” subscale of the OPS-NVI from the previously cited Delwel et al. (2018) [29] study, which had a sample of 348 dementia sufferers located in the Netherlands [31].

The Delwel et al. (2018) [29] study used a Dutch version of the OPS-NIV with the aim to assess the psychometric properties of the OPS-NVI as a screening tool for orofacial pain in people with mild cognitive impairment (MCI) or dementia. It used single and double observers (whenever possible) to score pain-related behaviours during rest, drinking, chewing and oral hygiene care. The expertise of the observers was not revealed although training in using the OPS-NVI instrument was given.

Whenever two observers were available, the interrater reliability per included behaviour item and observed pain presence of two observers was determined with the average positive agreement (PA), calculated by the formula: PA = 2a/(2a + b + c) and the average negative agreement (NA), calculated by the formula: NA = 2d/(2d + b + c), where a indicates a positive score for both observations, b and c indicate disagreement between the observations and d indicates a negative score for both observations. An average PA or NA below 60% was considered an insufficient score. The criterion validity of the OPS-NVI was determined by comparing the OPS-NVI pain observations with the reference standard: the reliable pain self-reports (MMSE score ≥ 14) [29].

The same dementia residents were observed in the de Vries et al. (2016) [31] study. Two observers (both sixth year dental students at the Academic Centre for Dentistry Amsterdam) used the OPS-NVI for 237 video clips of people with dementia in Dutch nursing homes during their meal to observe their behaviour and to estimate the intensity of orofacial pain [31]. Six weeks later, the same observers rated the video clips a second time. To establish the reliability of the “chewing” subscale of the OPS-NVI, the interobserver and intra-observer reliability were assessed by analysing the test–retest reliability for individual items of the instrument. The sum scores of the items per category and the interobserver and intra-observer reliability of the estimated pain score were also analysed. For all intra-observer reliability analyses, the *t*0-measurements of both observers were used [31].

The final study included in this evidence synthesis conducted by Van de Rijt et al. (2020) [30], was conducted across four UK residential settings based in London. The study included 128 people aged ≥ 65; 84 were diagnosed with dementia and could self-report oral pain symptoms, with a control group of 44 people without a diagnosis of dementia. Chi-squared tests, independent sample t-tests and Mann–Whitney U-tests (depending on data distribution) were used to compare demographics, oral health, orofacial pain and quality of life between the groups. To assess the validity of the OPS-NVI, the sensitivity, specificity and area under the receiver operating curve (AUROC) for each activity were calculated. The outcome of the self-report pain scale, the NRS, was used as gold standard [31].

### 3.3. Quality Assessment

Critical appraisal of the research studies enables a careful and systematic evaluation of the research to judge its value and trustworthiness before promoting as evidence-based practice. A number of tools are available to guide this process and can be adopted according to the design of the research. For example, CASP (https://casp-uk.net/what-is-critical-appraisal/ (accessed 14 September 2022)) has been developed and is commonly used to assess the quality of RCT’s, qualitative studies, economic evaluations and systematic reviews. Another developed by NICE (National Institute for Health and Care Excellence, https://www.nice.org.uk/process/pmg4/chapter/appendix-g-quality-appraisal-checklist-quantitative-studies-reporting-correlations-and (2012) (accessed 14 September 2022)) has developed a tool to assess the quality of correlation studies. Of the four retained studies for this review, two were correlation studies and two were systematic reviews and were thus assessed as follows:

Toxopeus (2016) [28]+
de Vries et al. (2016) [31] +
As validation studies, both were not UK based and there was no control group.
In terms of the systematic reviews.
Delwel et al. (2018) [29].
van de Rijt et al. (2020) [30] Both papers received maximum CASP score as the researchers adhered to all of the criteria for systematic reviews.

### 3.4. Results for Each Dental Pain Assessment Tool

The Toxopeus study [28] found substantial agreement for only two video fragments obtained during both sessions. For three fragments, the agreement was substantial during one session only. In addition, only three observers were able to provide consistent scores. For two of those, the various kappa values could be qualified as moderate to good (kappa = 0.39–0.74). Notably, all consistent scores pertained to dementia-related behaviours, not to orofacial pain/disability-related behaviours.

The study discussed how it was difficult to determine whether the videos of the residents during their mouth care routine showed pain adequately enough to be assessed or whether there was just an absence of pain. Pain-related behaviours might not have correctly represented behaviours associated with dental pain as it was difficult to differentiate between dementia-related behaviours and dental-pain-associated behaviours.

In the Delwel study [29], there was a high number of true negatives and a low number of true positives for pain observation compared with pain self-report, which was explained by the low pain presence (0–10%) in the sample. The items that seemed to be related to refusal or aggression, such as “resisting care,” “using offensive words,” “screaming/shouting” and “refusing prosthetics,” had a very low number of positive observations. This was suggested due to the sample being in the early stages of dementia and therefore less likely to show challenging behaviour. This population was chosen due to their ability to consent, which overcame ethical issues compared with studying people with later stages of dementia who are unable to consent.

When examining the cross-tabulation of the pain self-report of participants with a valid self-report (MMSE 14 or higher) and the oral health examination, many participants seemed to have potential painful conditions, such as ulcers, tooth root remnants, coronal caries and root caries, but did not express pain during the pain self-report. The authors suggested this might be due to the use of pain medication playing a role in the under reporting of pain. In addition, the pain self-report was a cross-sectional measurement and the mentioned conditions may just cause pain and/or inflammation at other moments. Therefore, a regular oral examination by care providers and oral hygiene care professionals remains necessary. A major limitation reported was there was the possibility of bias of the observers due to observation of the different activities directly after each other.

In the De Vries [31] study, it used the “chewing” subscale of the OPS-NVI instrument to assess pain in same population of dementia residents from the Delwel study [29] to record possible dental pain during their meal. This study found there was a discrepancy between the different observers: the intra-observer reliability of observer 1 ranged from fair-to-good to excellent (0.76–0.85), whereas the intra-observer reliability of observer 2 ranged from poor to fair-to-good (0.49–0.66). In the process of assessing the interobserver and intra-observer reliability, it was found that a total of ten items could be excluded from this subscale of the OPS-NVI, which the authors suggested makes the instrument more concise and easier to use. However, the need for further assessment of the reliability of the OPS-NVI in subjects with more severe orofacial pain is demonstrated by both the Delwel [29] and De Vries [31] studies.

The Van de Rijt [30] study using the OPS-NVI to assess dental pain in residents with dementia demonstrated pain was present in 48.8% (95% confidence interval (C.I.) 36.1–50.7) of residents with dementia. Self-reported orofacial pain was present in 37.8% (95% C.I. 20.4–53.7) of residents with dementia and in 14.8% (95% C.I. 0.5–30.4) residents without dementia. Orofacial pain was significantly more prevalent in residents with dementia than those without (OPS-NVI; *p* = 0.002, self-report; *p* = 0.04). Having a soft diet, xerostomia, being dentate, and poor oral hygiene in dentate residents were significant predictors of orofacial pain in residents with dementia.

The sensitivity of the OPS-NVI was 66.5–100%, the specificity was 76.9–100%, the AUROC was 0.794–1.0 and the accuracy was acceptable to outstanding.

Limitations to this study were the target sample size of 94 participants per group not being met and the ratio of residents with and without dementia being unequal (over two-thirds have dementia). Therefore, it is important to acknowledge that the control group of people without dementia may not have been ‘typical’ UK care home residents.

### 3.5. Findings on Experiences of Assessing Dental Pain

Although the cross-sectional exploratory qualitative study did not discuss informal carer experience of using a specific dental pain assessment instrument, it did discuss how the carers assessed pain and therefore is still worthy of mention.

The study by Newton et al. (2018) [32] included 35 ‘informal carers’ (spouse, sibling or friend).

Five focus groups were conducted that asked questions about dental pain assessment, carrying out oral hygiene, accessing help, and what carers felt they needed.

The signs and symptoms that the carers felt would indicate oral pain were largely based on similar physiological and behavioural indicators used by healthcare professionals. Being with the person daily helped identify pain that was noticed during oral hygiene habits or eating. These included changes in behaviour relating to being off their food and losing weight quickly, wincing and cupping the face, sleep disturbance/not relaxing, bad breath, drooling, anger, being ‘jumpy’ and refusing dentures. Carers’ accounts also described how, and why, they were often best placed to see the signs and symptoms of oral pain and discomfort as they cared for the person all day.

## 4. Conclusions and Recommendations

Our systematic review demonstrates that there has been very little research into oral health and/or dental pain in adults with dementia. The studies that have been carried out have tended to be reviews or correlation studies—but it seems that the recommendations have yet to be taken forward. Identifying pain in older adults with dementia remains challenging [33,34] and there have been many attempts at developing scales to identify and measure pain [12] and yet we are aware that these are not universally applied [35]. The work of Newton et al. (2018) [32] does suggest that carers are best placed to advocate for the individual with oral health needs as they spend most time with the individual, but the care home setting is busy and stretched for staff so this may not be feasible. In recent years, the COVID-19 pandemic has impacted significantly upon the work of care home staff and highlighted the importance of acknowledging the risks of cross contamination. Dental hygiene is an important factor that must be recognized as potentially causing cross infection where mouthcare devices should be single use and dedicated to individual use only. Similarly, access to dental services has been highlighted as impacted during the pandemic, particularly in relation to emergency care [35,36]. We need to explore new ways of identifying dental caries and pain in the care home resident and look for solutions to support care home staff in delivery of dental care. Our recent stakeholder group has brought together dentists and care home staff to begin this journey, which will be reported in a separate paper.

## Supplementary Materials

The following supporting information can be downloaded at: https://www.mdpi.com/article/10.3390/geriatrics7050103/s1, S1: PRISMA 2009 Checklist.
